# The gut-immune-brain axis in neurodevelopment and neurological disorders

**DOI:** 10.20517/mrr.2022.11

**Published:** 2022-08-17

**Authors:** John Chulhoon Park, Sin-Hyeog Im

**Affiliations:** ^1^Department of Life Sciences, Pohang University of Science and Technology, Pohang 37673, Republic of Korea*.*; ^2^Institute for Convergence Research and Education, Yonsei University, Seoul 03722, Republic of Korea.; ^3^ImmunoBiome Inc., POSTECH Biotech Center, Pohang 37673, Republic of Korea.

**Keywords:** Gut-brain axis, neuroimmunology, neuroinflammation, blood-brain barrier, microbiota, probiotics, autism spectrum disorder, Alzheimer’s disease

## Abstract

The gut-brain axis is gaining momentum as an interdisciplinary field addressing how intestinal microbes influence the central nervous system (CNS). Studies using powerful tools, including germ-free, antibiotic-fed, and fecal microbiota transplanted mice, demonstrate how gut microbiota perturbations alter the fate of neurodevelopment. Probiotics are also becoming more recognized as potentially effective therapeutic agents in alleviating symptoms of neurological disorders. While gut microbes may directly communicate with the CNS through their effector molecules, including metabolites, their influence on neuroimmune populations, including newly discovered brain-resident T cells, underscore the host immunity as a potent mediator of the gut-brain axis. In this review, we examine the unique immune populations within the brain, the effects of the gut microbiota on the CNS, and the efficacy of specific probiotic strains to propose the novel concept of the gut-immune-brain axis.

## INTRODUCTION

The last few decades have seen exponential growth in studying human commensal microbes and their impacts on our physiology. A few milestone publications established the ability of gut microbes to alter neurological functions, birthing what is today one of the most prominent new fields in biology, the microbiota-gut-brain (gut-brain) axis^[1]^. Since then, numerous clinical and animal studies have revealed the link between gut microbiota and neurological outcomes and their capacity to alleviate them therapeutically^[2,3]^. Much of this progress has been driven by behavioral analyses of germ-free (GF) mice that lack microflora and antibiotic-treated (ABX) mice, which exhibit abnormal behaviors including reduced sociability, fear extinction learning, and anxiety compared to their specific-pathogen-free (SPF) counterparts^[4-7]^. Accordingly, probiotics have gained attention as potential therapeutic modulators, such as *Lactobacillus reuteri *ATCC PTA 6475 or *Lactobacillus acidophilus *Rosell-11, which can prevent autism spectrum disorder (ASD)-associated behaviors in both mice and human ASD patients^[8-10]^. Moreover, a recent study revealed that the fecal microbiota transplantation (FMT) of human ASD patient feces into mice induced ASD-associated behaviors in their offspring, hallmarking the centrality of gut microbes in neurological disorders^[11]^.

In gut microbiota studies, host immunity is often a critical component that links gut microbes with host physiology. Up to 1 × 10^14^ microbes from over 7000 strains compose the human gut microbiota, which closely interacts with the gastrointestinal immune system to maintain homeostasis^[12]^. Innate immune populations such as dendritic cells and macrophages must discern between pathogenic and commensal microbial signals to elicit a protective or inflammatory response by adaptive immune cells^[12,13]^. Beyond mediating local immunogenic tolerance, the gut-immune crosstalk can facilitate systemic immune reactions, as evidenced by the ability of gut microbe-induced regulatory T cells (Tregs) to prevent autoimmune disorders including colitis and experimental autoimmune encephalomyelitis (EAE)^[14,15]^. Conversely, dysbiosis and the accumulation of specific bacterial taxa in the gut can directly exacerbate autoimmune diseases such as multiple sclerosis by elevating proinflammatory Th1 and Th17 responses and downregulating IL-10 producing Tregs^[16]^. In this manner, host immunity is a crucial mediator within the gastrointestinal tract and the gut-brain axis.

In the central nervous system (CNS), the brain contains a tight immunological microenvironment that regulates neurological outcomes^[17]^. While microglia have traditionally been considered the main brain-resident immune population, several innate and adaptive immune cells are recognized to reside and function within the brain, including CD4 and CD8 T cells^[18]^. These brain resident lymphocytes can directly interact with neurons through cytokines and cell surface receptors and indirectly through modulating astrocyte and microglia functions^[17,19,20]^. The rising evidence in these fields indicates the potential for gut microbes to regulate brain-resident immune populations, resulting in altered neuronal activity. This review summarizes the current knowledge in each area within the gut-immune-brain axis and highlights their interconnected nature. More significant research into this axis may provide a deeper understanding of the mechanisms behind neurological disorders, opening up a new avenue for unique therapeutic approaches.

## TYPES AND ROLE OF IMMUNE CELLS IN THE CENTRAL NERVOUS SYSTEM

### Blood-brain barrier

**Figure 1:**
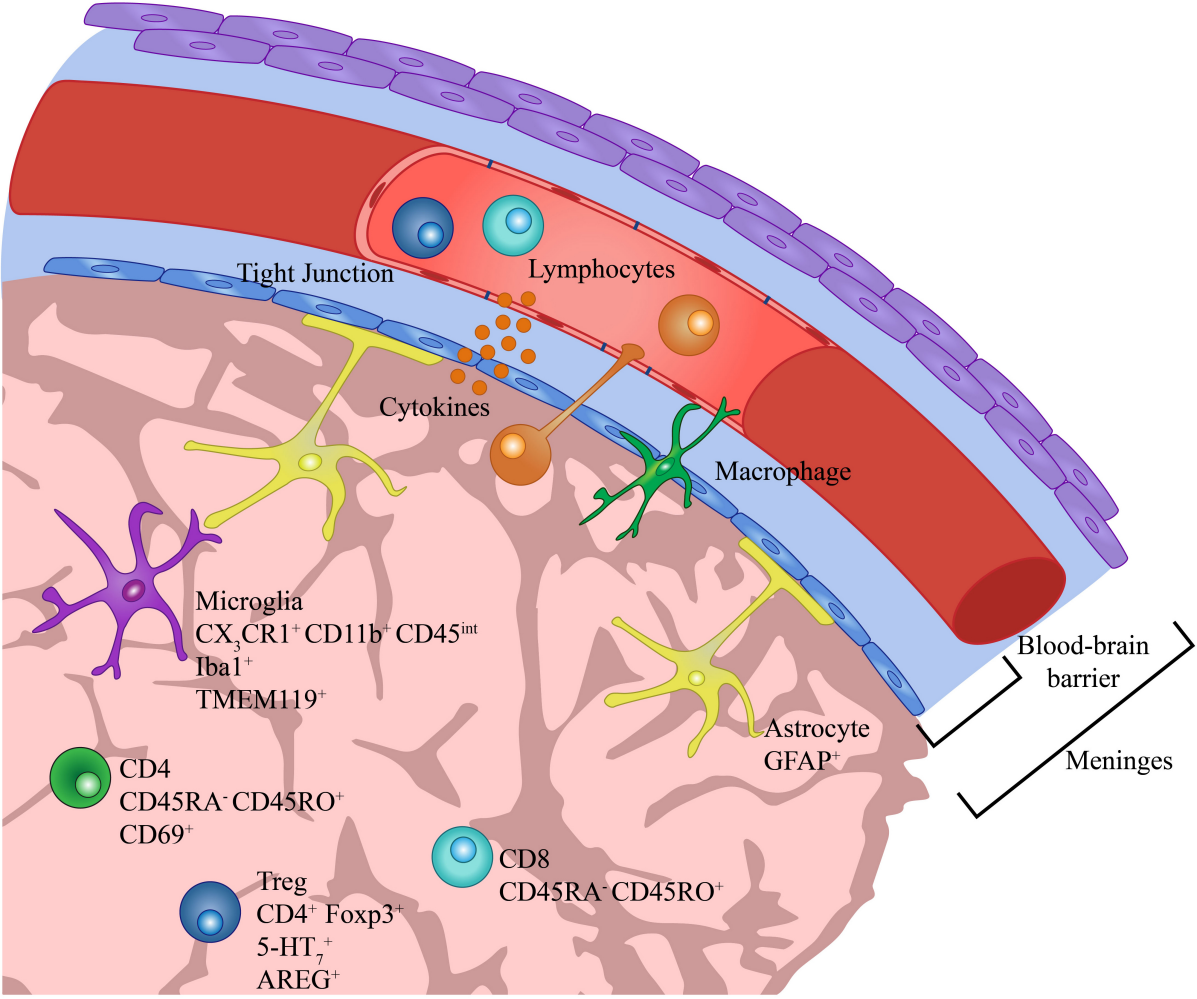
Immune populations of the brain microenvironment. The blood-brain barrier regulates the influx of peripheral immune populations into the brain. However, recent findings have also identified brain-resident lymphocyte populations in diseased and homeostatic brain parenchyma.

Classical neuroimmunology was limited by the perception of microglia as the sole immune population due to the blood-brain barrier (BBB), which seemed to isolate the brain microenvironment from the rest of the peripheral immune system. However, recent studies unveil that peripheral antigens, cytokines, and metabolites alter the BBB integrity and cross over to regulate brain-resident immune cell functions^[21]^. The BBB is a selective semipermeable barrier composed of endothelial cells of blood vessels, pericytes, glial cells, and extracellular matrix that protect the brain and maintain homeostasis of the microenvironment^[22]^. Tight junctions, transporters, and transcytosis regulate the influx of peripheral molecules across the BBB, and peripheral immune cells can be trafficked through chemokine signaling and cell surface adhesion molecules^[22,23]^. The modern discovery of distinct immune niches within the brain provides new insight into the multifaceted regulation of neurodevelopment and neurological disorders [Figure 1].

### Innate immune populations

Microglia are the tissue-resident macrophages of the brain and are the fundamental regulators of immune surveillance, synapse pruning, and homeostatic maintenance of the central nervous system^[24]^. CX3CR1^+^CD11b^+^CD45^int^ microglia of the CNS can be distinguished from infiltrating macrophages by the microglia-specific TMEM119 marker and activation by Iba1 expression levels^[25,26]^. As the distribution of microglia and macrophage populations across the brain differ, each subset constitutes unique roles during homeostasis and disease. Microglia can be derived from myeloid progenitors in the embryonic yolk sac or bone marrow-derived Ly6C^hi^ monocytes that cross the BBB^[27,28]^. Under homeostasis, most microglia populations are yolk-sac progenitor-derived endogenous microglia that regulate neuronal growth, organization, synaptic maturation, and pruning^[29,30]^. However, during certain diseased conditions, a high influx of peripheral bone marrow-derived monocytes can occur through the BBB, especially during neuro- and systemic inflammation^[31]^. Mainly, sustained systemic inflammation can cause microglia to initiate BBB leakage, either through the production of proinflammatory cytokines or phagocytosis of astrocyte end-feet^[32,33]^. Neurological stress, such as repeated social defeat stress, has also been shown to recruit peripheral monocytes into the brain, following which their IL-1β production causes stress-induced anxiety^[34]^. Many studies on the functional role of microglia in neurological disorders have utilized selective targeting and ablation of microglia using the colony-stimulating factor 1 receptor (CSF1R) inhibitors such as BLZ9445, PLX3397, and PLX5622, which can cross the BBB to arrest microglial and macrophage cell growth^[35-37]^.

### Astrocytes

Apart from microglia, astrocytes compose the major population of glial cells within the CNS, interweaving throughout the entire brain to facilitate a wide array of functions from regulation of synaptic transmissions and neural circuits to mediating immune insults to the CNS. The gravity of astrocytes in maintaining CNS homeostasis is well illustrated through numerous astrocyte-ablation experiments. For instance, transgenic ablation targeting the astrocyte-specific marker glial fibrillary acidic protein (GFAP) has demonstrated significant neurodevelopmental deficits, particularly in the cerebellum, resulting in perturbation of cellular layers, neuronal degeneration, and severe ataxia^[38,39]^. Ablation of astrocytes during pathological conditions further demonstrates their necessity. In Alzheimer’s disease (AD), pharmacological or transgenic depletion of astrocytes results in the increased amyloid-β (Aβ) accumulation with reduced clearance mechanisms, highlighting the protective role of astrocytes in AD progression^[40,41]^. A similar neuroprotective capacity for astrocytes was demonstrated in a transgenic astrocyte ablation model of multiple sclerosis, EAE, where the absence of astrocytes exacerbated CNS inflammation through the infiltration of macrophages, T cells, and neutrophils^[42]^. 

Indeed, astrocytes maintain a unique niche within the CNS where they translate between the immunological and neuronal activities of the microenvironment. Upon stimulation, astrocytes undergo transcriptional and physiological changes collectively known as reactive astrogliosis that primes them to respond to CNS insults^[43]^. One of the hallmark signatures of reactive astrogliosis is the upregulation of GFAP expression by astrocytes, which can occur in response to immunological stimuli such as TGFβ, IL1β, IFNγ, and IL6 from microglia and leukocytes, LPS and metabolites from the microbiota, and mechanical stimuli from the CNS microenvironment^[44-46]^. The resulting responses from astrocytes are as diverse as the stimuli for reactive astrogliosis. Microglia-astrocyte communication is one of the foundational immune crosstalk within the CNS to modulate neuroinflammation and maintain homeostasis. This bidirectional relationship is demonstrated in numerous neuropathologies. One recent example has been the clearance of Aβ by microglia in response to astrocytic IL3 during AD^[46-48]^. Conversely, microglial signals such as TGFα and VEGF-B have also been shown to control astrogliosis during EAE^[46-48]^. Furthermore, astrocytes serve a critical role in T cell modulation in the context of neuroinflammation. Astrocytic expression of CTLA4, CD39, and CD73 can induce T cell suppression. At the same time, the production of cytokines and chemokines such as IL12, IL23, IL1β, IL6, TNFα, TGFβ, CXCL1, and CCL2 can result in T cell recruitment and polarization towards proinflammatory phenotypes^[49,50]^.

### Lymphocytes

The role of lymphocytes in neuroimmunology has long been a puzzling topic. The brain is believed to be an immune-privileged site, with many classical neurobiologists expressing skepticism towards the idea of lymphocyte residence within the CNS. However, T cell-driven neuroinflammatory disorders such as EAE, an animal model for multiple sclerosis (MS), clearly hinted at the functional importance of lymphocytes in the neuropathology of the CNS. While still a highly contested subject, numerous studies in the recent decade have identified the presence of lymphocytes within the CNS - not only during neuropathology but even in healthy brains, pointing to their potential roles in maintaining CNS homeostasis^[18]^. 

### Meningeal T cells

Meningeal T cells are perhaps the well-accepted group of CNS-resident lymphocytes. CyTOF mass cytometry analysis of brain-resident immune cells in homeostatic C57BL/6 mice reveals that most CD45^high^ cells are located within the meninges and the choroid plexus compared to the parenchyma^[18]^. Cytokines released by these meningeal T cells contribute to neuropathology. During EAE, meningeal Th17 cells drive neuroinflammation by producing proinflammatory IL17A, further recruitment of leukocytes into the meninges, and induction of follicular meningeal B cell activation^[51,52]^. A similar phenomenon is recorded in ischemic brain injury models where T cell infiltration and IL17 production drives cerebral infarction^[53,54]^. IL17A can also be directly recognized by IL17RA expressing hippocampal neurons, which induce dysfunctional hippocampal long-term potentiation and significant cognitive impairments^[55]^.

Beyond neuroinflammation, meningeal T cells can regulate healthy neural connectivity, especially in the context of social behaviors through IFNγ signaling, as their depletion results in reduced sociability in the three-chamber sociability assay^[56]^. Furthermore, meningeal γδ T cells have been shown to modulate homeostatic behaviors by producing IL17A to trigger anxiety-like behaviors in mice^[57]^. Interestingly, these IL17-producing γδ T cells may have a double-sided capacity, as they have also been shown to be necessary for standard short-term memory and cognition, implicating the complex and multifaceted role of meningeal T cells in healthy and inflamed CNS microenvironments^[58]^. 

### Parenchymal T cells

Several publications in recent years have verified the existence of T cells within the parenchyma of healthy and diseased brains of both mice and humans through immunofluorescence imaging and flow cytometric analysis^[17,19,59]^. In the healthy mouse brain, while CD4^+^ T cells are the richest within the meninges, more than 75% of brain-associated CD4^+^ T cells reside within the parenchyma^[19]^. Flow cytometric analysis of human brain white matter further identified differentiated CD45RA^-^ CD45RO^+^ CD4^+^ and CD8^+^ T cells that highly express CD69, representing tissue-resident memory T cells^[17,60]^. The expression of tissue-homing receptors such as CX3CR1, CXCR3, and CCR5, the lack of lymph node-homing CCR7, and blood-brain barrier-crossing CD49d support the local resident, rather than infiltrating, nature of these T cells^[17,59,60]^. This is also supported by experiments where large quantities of CD45-tagged CD4^+^ T cells are identified within the brain following intravenous injection with CD45-fluorescence-labeled antibodies^[19]^. Of note, these brain-resident T cells expressed high levels of PD1 (programmed death-1) and CTLA4 (cytotoxic T lymphocyte-associated antigen-4), pointing towards a potential neuroprotective mechanism that suppresses inflammatory immune activation^[17]^. Indeed, numerous neuroinflammatory conditions can be exacerbated by T cell-sourced effector molecules, such as granzyme-b and IFNγ^[61-63]^. Apart from their roles during neuroinflammation, the importance of brain-resident T cells can be demonstrated by the failure of microglial maturation in the absence of CD4^+^ T cells which results in abnormal neuronal synapses and impaired learning behavior^[19]^.

### Brain-resident Tregs

In classical immunology, Tregs are the fundamental gatekeepers of immune homeostasis. Remarkably, recent studies have identified small populations of resident Tregs within the brain^[20,64]^. Of the roughly 2000 CD4^+^ T cells that can be quantified within the healthy mouse brain, Tregs compose ~150 of them^[19]^. While Tregs are yet to be quantified or functionally analyzed in human brains, analysis of rat brains has also determined around 900 Tregs within the cerebellum, composing about 15% of the local CD4^+^ T cells^[64]^. This suggests that, although few in number, brain-resident Tregs are a conserved population of functionally active T cells within the healthy brain. The higher expression of activation markers such as ICOS, CTLA4, KLRG1, CD103, and CD69 compared to peripheral Tregs attributed to an activated and memory phenotype, which is supported by their highly suppressive capabilities during the *in vitro* Treg suppression assay and *in vivo *LPS-induced neuroinflammation^[19,64]^. Furthermore, neuroinflammatory conditions such as murine cytomegalovirus or neuromyelitis optica spectrum disorder necessitate Tregs to attenuate leukocyte trafficking and proinflammatory cytokine production within the brain^[65,66]^. Tregs have also been demonstrated to interact and restrict glial populations of the CNS. During ischemic stroke, the massive accumulation of Tregs into the brain effectively suppresses reactive astrogliosis and microglial IL6 production^[20]^. Interestingly, IL2 and IL33 from astrocytes are necessary for Treg maintenance and amplification within the brain, underscoring a unique bidirectional relationship between these two populations^[20,64]^. Emerging evidence that Tregs can facilitate non-canonical roles such as organ-specific tissue repair in zebrafish highlight undiscovered capacities for these cells within the CNS^[67]^.

## IMPACT OF THE GUT MICROBIOTA ON THE BRAIN

### Microbiota alters neurological development and disorders

The notion of an isolated brain during neurodevelopment and neurological disorders is challenged not only by the presence of unique residential immune populations but also by gut microbes and their effector molecules on the CNS. In the case of autism spectrum disorder (ASD), a neurodevelopmental disorder that affects 1 in 44 children in America^[68]^, over a thousand gene mutations are potentially linked to the disorder and was considered a solely genetically-driven disorder^[69]^. However, the high comorbidity between ASD and gastrointestinal disorders suggests the role of gut microbiota in ASD pathology. Microbiome sequencing studies support this among ASD patients that identify trends in their microbial signatures, such as increases in *Lactobacillus*,* Clostridium*, and* Bacteroides *genus and decreases in *Bifidobacterium*^[70,71]^. The differences observed in ASD patients’ gut microbiomes and the spectral nature of behavioral phenotypes in ASD may reveal an intricate association between behaviors, neurological components, and causal mechanisms. A recent study dissected the mechanisms driving hyperactivity and social deficits - two common behavioral abnormalities associated with ASD in the *Cntnap2^-/-^* mouse model for ASD^[72]^. Through a series of eloquent breeding techniques between *Cntnap2^-/-^* and *Cntnap2^+/+^* mice, Buffington *et al.* defined the genetic causal for hyperactivity and the gut microbiome’s ability to mediate sociability^[72]^. Treatment of these mice using probiotic *L. reuteri* ATCC PTA 6475 supported their findings through which the antisocial trait of this genetic mouse model was normalized to that of wild-type neurotypical mice, yet remaining insufficient to resolve hyperactivity^[72]^. 

Recent studies provide a close correlation of gut microbiota with murine sociability. GF mice have been a critical tool in identifying the link between gut microflora and neurodevelopment. GF or antibiotic-treated (ABX) C57BL/6 mice often display spatial, learning, and contextual memory impairments during behavioral tests such as the Morris water maze or fear conditioning and extinction learning assays^[5,73]^. Such wild-type mice that lack microbiota have also been well recorded to have severe social deficits, abnormal motor functions, and elevated levels of anxiety^[5,6,73-75]^. However, it is necessary to note that institutional animal facilities and experimental techniques may influence assays for anxiety, as results on anxiety and motor activity between GF and specific pathogen-free mice are contested, with some data showing elevated anxiety and decreased motor activity in GF mice, and others are demonstrating the opposite^[6,73]^.

### Impact of gut microbes and their metabolites on the brain

**Figure 2:**
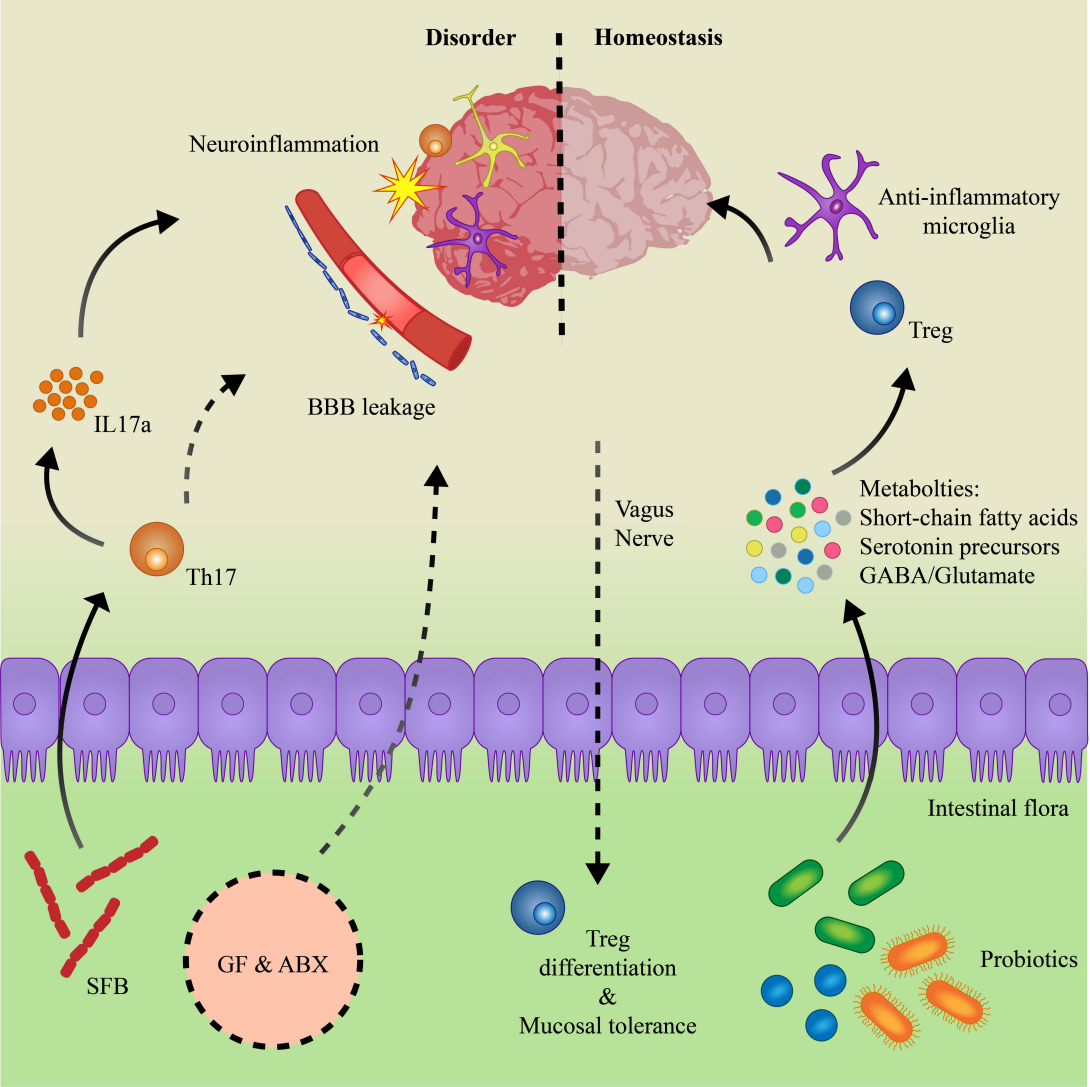
Model of the gut-immune-brain axis. Microbes can directly shape the brain microenvironment through their metabolites or indirectly through modulation of the host immune system. The absence of microflora in germ-free (GF) or antibiotics-treated (ABX) mice have been demonstrated to induce blood-brain barrier (BBB) leakage^[85,86]^. Segmented filamentous bacteria (SFB) can stimulate the production of IL17a by Th17 cells, which drives neuroinflammation^[105,108-110]^. On the other hand, beneficial probiotics such as *L. reuteri *ATCC PTA 6475 or *B. longum *subsp.* infantis* DSM 24737 can protect from neurological disorders through metabolic pathways and the induction of immune homeostasis. Brain-to-gut signals through the vagus nerve control colonic regulator T cell (Treg) populations to regulate gastrointestinal homeostasis.

The mechanism behind these behavioral changes in GF and ABX mice can be organized into two pathways: the direct effect on the CNS by the microbes and the immune-mediated [Figure 2]. First, it has been demonstrated that the absence of gut microflora can perturb the neural microenvironment. GF mice have higher levels of noradrenaline, dopamine, and serotonin in the striatum, which coordinates motor activities, and altered synaptic plasticity as measured by expression of nerve growth factor-inducible clone A (NGFI-A), brain-derived neurotrophic factor (BDNF), and postsynaptic density protein-95 (PSD-95)^[6]^. Transcriptomic analyses of brain regions also identify significant shifts in neuronal pathways, particularly related to synapse assembly and organization, calcium signaling pathways, and axonogenesis^[5,6,74]^. Furthermore, GF and ABX mice have elevated c-FOS expression, a marker for neuronal activation, within the basolateral amygdala, which may explain the alterations in stress or fear-associated behaviors^[4,5]^.

Interestingly, in many of these studies, recolonization of GF or ABX mice with specific microbes or whole microbiomes of donors elucidates a metabolomic mechanism by which the gut microbiota remodel the brain. For instance, social deficits in GF mice can be reversed by mono-colonization with *Enterococcus faecalis* ATCC 19433, as can whole microbiome transplant from healthy human and mouse donors^[4,9,11]^. Other abnormal behaviors such as repetitive behavior and anxiety have also been alleviated by *Bifidobacterium dentium* ATCC 27678 mono-colonization in GF mice through the regulation of serotonin signaling^[76]^. Indeed, *Clostridium*,* Burkholderia*,* Streptomyces*,* Pseudomonas*, and *Bacillus *genera are highly enriched for tryptophan metabolism and correlate with neurological disorders associated with serotonin^[77]^. The gut microbiome sources many essential metabolites regulating host physiological states, including neurodevelopment. This is apparent by assessing the gamma-aminobutyric acid (GABA)/glutamate cycle in mice with altered microbiomes. GF mice transplanted with fecal microbiomes from schizophrenia patients demonstrate disease-associated behavioral abnormalities driven by reduced glutamate and elevated glutamine and GABA levels within the hippocampus^[78]^. Similarly, in a mouse model for epilepsy, the dietary intervention was able to protect mice from seizures by increasing the GABA/glutamate ratio in the hippocampus^[3]^. Treatment with *Akkermansia muciniphila* ATCC BAA835 and *Parabacteroides merdae *ATCC 43184 revealed a mechanism driven by bacterial cross-feeding between these microbes under the ketogenic diet^[3]^. *Bifidobacterium adolescentis *has also been identified as a GABA producer, with the *B. adolescentis *PRL2019 and HD17T2H strains demonstrating abilities to elevate GABA levels *in vitro *and *in vivo *rat models^[79]^. Several bacterial strains can produce glutamate, including *Lactobacillus plantarum *and* Lactobacillus paracasei*, and it has been hypothesized that the gut microbiota can regulate gamma-glutamylated amino acids^[3,80]^. The expression of GABA receptors within the CNS can also be modulated, as demonstrated by *Lactobacillus rhamnosus *JB-1 treatment in mice models for anxiety and despair^[81]^. The neurological changes among GF and ABX mice and the association between gut microbiota and neurotransmitters attest to a direct pathway in which gut microbes influence the CNS.

### Gut microbiota and the blood-brain barrier

Barriers are the first regulation line separating the gut microbiota and the rest of the body. Dysregulation of the gut microbiota is identified as an etiopathology for irritable bowel disease and results in gut barrier dysfunction and inflammation^[82,83]^. The disruption of gut barriers allows more significant exposure to microbial metabolites and cytokines that drive systemic inflammation^[84]^. Similarly, increasing evidence suggests that the gut microbiota may be responsible for regulating BBB permeability, thus facilitating immune insult to the brain. The absence of gut microbes in GF and ABX mice has been shown to induce increased BBB permeability marked by decreased expression of tight junction proteins claudin-5 and occluding, resulting in cognitive disability^[85,86]^. Probiotic preparations such as the ProBiotic-4, which contains strains belonging to the species *Bifidobacterium animalis *subsp. *lactis*,* Bifidobacterium bifidum*, *Lactobacillus casei*, and *L. acidophilus*, have also been shown to protect BBB integrity and improve memory deficits in murine Alzheimer’s disease model^[87]^. Microbiota-derived metabolites may drive changes in barrier integrity, as mono-colonization with specific short-chain fatty acid (SCFA)-producing bacteria such as *Clostridium tyrobutyricum *DSM 2637 (butyrate-producing) or *Bacteroides thetaiotaomicron* (acetate and propionate producing) in GF mice protect from BBB permeability^[85]^. Additionally, commensal microbes can break down dietary methylamines into trimethylamine, which is converted into trimethylamine-N-oxide that can enhance BBB integrity and protect from neuroinflammation^[88]^.

Gut microbiota-derived molecules can alter the BBB permeability and cross it to interact with brain-resident immune cells directly. One example is lipopolysaccharides (LPS) of Gram-negative bacteria, which have been shown to cross into the BBB using transport mechanisms involving lipoprotein receptors^[89]^. Within the brain, LPS can bind to toll-like receptor (TLR) 4 on microglia and astrocytes to induce a proinflammatory activation^[90,91]^. A recent study demonstrated this capacity using a rat model of EAE, in which LPS could cross the BBB to shift microglia towards a type 1 IFN response, protecting them from the type 2 IFN-mediated EAE^[92]^. While the full extent of microbial metabolites’ impact on the BBB is yet to be understood, emerging findings allude to their interaction as a critical component during neurological disorders.

### The gut microbiota on microglia

Neuroimmune populations are potent regulators between the gut and the brain. As with many neuroimmune events, microglia are the key mediator in reshaping the brain microenvironment in response to microbial metabolites. Alzheimer’s disease (AD) is a neurodegenerative disorder often defined by the accumulation of Aβ and tau proteins in the brain. Although AD has traditionally only been studied from a neurological perspective, recent works implicate an immune and microbial basis in the development of the disorder. GF and ABX mouse models for AD are protected from Aβ plaque aggregation and neuroinflammation^[93,94]^. Furthermore, transgenic AD mice compose a distinct microbiome from their wild-type counterparts, with AD-associated microbiomes shifting with age and disease progression^[2]^. This is expounded in an experiment during which transplant of AD patient’s microbiota into transgenic AD mice exacerbates the pathology, highlighting an AD-specific microbiome behind the disorder^[95]^.

In all of these individual studies, the microglia is the mechanism tying the microbiota with AD. Microglia of GF and ABX-treated AD mice have better recruitment and uptake of Aβ deposits, as well as reduced inflammatory phenotypes^[2,94]^. This may be attributed to microbial metabolite profiles in AD patients. For instance, metabolomic analysis of serum and fecal samples from AD and healthy individuals have identified reduced microbial SCFAs, including formic acid, acetic acid, propanoic acid, butyric acid, 2-methylbutyric acid, isovaleric acid, and valeric acid in AD patients^[96]^. Significant changes within the serotonin pathways were also detected, with reductions in numerous tryptophan derivatives in AD patients^[96]^. Tryptophan metabolism, the precursor for serotonin, is a crucial part of microglial activation, as its derivatives are ligands for the aryl hydrocarbon receptor (AHR) expressed on microglia and astrocytes^[97]^. Indeed, microbial metabolism of tryptophan has been directly shown to induce microglia activation through the AHR to block proinflammatory phenotypes in astrocytes, indicating how microglia translate the gut microbiota to impact the brain microenvironment^[48]^. 

Additionally, bile acids are beginning to be identified as another messenger between the gut microbiome and the brain^[98]^. Bile acids are predominantly formed in the liver, but the gut microbiota is required to metabolize primary bile acids into secondary bile, which has been shown to impact microflora composition^[98,99]^. Primary and secondary bile acids can cross the BBB and interact with the farnesoid receptor and Takeda G-protein receptor 5 (TGR5), the latter of which is also expressed by microglia^[99,100]^. Interestingly, TGR5 signaling in microglia has been shown to attenuate IL1β, IL6, and TNFα levels during neuroinflammation^[101]^. In animal studies, the conjugated bile acid tauroursodeoxycholic acid has also demonstrated restrained glial activation during AD, resulting in reduced Aβ plaque^[102]^.

### T cells within the gut-brain axis

Adaptive immunity is also at the core of many microbiota-associated brain disorders. One of the best examples is demonstrated during the maternal immune activation (MIA) model for ASD. Infections and severe inflammatory conditions during pregnancy are the risk factors for neurodevelopmental disorders, including schizophrenia and ASD^[103,104]^. The MIA mouse model mimics this phenomenon by triggering an IL17A-driven immune response following injection of synthetic double-stranded RNA polyinosinic:polycytidylic acid (poly[I:C]), which induces irregular neurodevelopment and ASD-associated behavioral abnormalities^[105]^. This model is suggested to be microbiota-dependent, as MIA offspring are marked by gut microbiota dysbiosis and high levels of the microbial metabolite 4-ethylphenyl sulfate, which drives anxiety^[106]^. The microbial role in MIA is further supported by ABX treatment, which protects MIA offspring from behavioral deficits^[107]^. Furthermore, Kim *et al. *demonstrate that the MIA model is only functional in C57BL/6 mice from Taconic Biosciences but not in those from Jackson Laboratories - a difference derived from the presence of the commensal microbe segmented filamentous bacteria (SFB)^[108]^. SFB can increase the IL17a pool by stimulating Th17 cells in the intestines, invoking neuroinflammation through reception by IL17Ra expressing neurons, microglia, and astrocytes^[105,108-110]^. Additionally, MIA-associated microbes can enhance IL17a production in offspring following *Citrobacter rodentium *infection, supporting a close link between maternal immunity and microbiota, consequent offspring neurodevelopment, and immune activation^[111]^. In neuroimmunology, no study yet exists on the effect of gut microbes on brain-resident Treg cells. However, the fact that Tregs are essential suppressors of neuroinflammation and that brain Tregs express unique genes related to the nervous system, such as neuropeptide Y, serotonin receptor 7 (encoding 5-HT_7_), and amphiregulin (AREG), indicate that they may be sensitive to modulation by microbial metabolites^[20]^. Future studies are necessary to elucidate whether the gut microbiota can regulate neurological disorders by activating brain Tregs.

## NEURAL REGULATION OF THE GUT MICROENVIRONMENT

The gut-brain axis constitutes a bidirectional exchange in which signals from the brain can also exert changes on the gut microbiome. One of the central pathways for this interaction is mediated by the vagus nerve (VN), which constitutes both afferent and efferent neurons and innervates the digestive tract^[112]^. Gut microbiota-to-brain signaling through the VN is well recorded, with microbial metabolites, gastrointestinal inflammation, and gut hormones altering brain functions^[112-114]^. On the other hand, evidence of brain regulation of the gut microenvironment is just starting to emerge. These studies have identified the intestinal immune system as a mediator of neural signals. Intestinal immune populations discern between commensal and pathogenic microbes to maintain immune tolerance and mutualism within the intestinal tract^[13]^. Tregs are at the heart of host-microbe immunity and suppress maladaptive immune activation towards commensal microbes. This ability is now attributed to the control of peripheral Tregs by a gut-brain arc, in which hepatic vagal nerves send sensory input from the gut microenvironment to the brainstem and return to the enteric neurons to induce colonic Treg proliferation^[115]^. Two mechanisms by which enteric neurons associate with Tregs have been proposed. The first identified intestinal antigen-presenting cells that communicate with enteric neurons via muscarinic acetylcholine signaling, as demonstrated by the reduction of colonic Treg cells in mice genetically lacking muscarinic acetylcholine receptors^[115]^. Another study corroborates this idea but points towards a direct interaction between enteric neurons and Tregs within the colon lamina propria^[116]^. Yan *et al.* identified Tregs residing adjacent to enteric nerve fibers that secrete IL6 to suppress RORγ^+^ microbe-responsive Treg proliferation^[116]^. This neuron phenotype is controlled by microbes that induce RORγ^+^ Tregs, suggesting an intricate gut-immune axis reconciled by the brain^[116]^. Specific brain regions are now recognized to be capable of memorizing and retrieving immunological events, including colitis, further highlighting the role of the CNS in maintaining gastrointestinal homeostasis^[117]^. Finally, neurological distress such as chronic stress and depression are well observed to cause gastrointestinal dysregulation through the hypothalamic-pituitary-adrenal axis, in which endocrine signals can shift the microflora balance^[118]^.

## PROBIOTICS-BASED THERAPEUTICS FOR NEUROLOGICAL DISORDERS TARGETING NEUROIMMUNE CELLS

With the emergence of the gut-brain axis, many have turned to probiotics as potential therapeutic candidates for neurological disorders (a summary of probiotics used in animal and clinical studies can be found in Table 1). Probiotics are defined by the FAO/WHO as “live microorganisms which when administered in adequate amounts confer a health benefit on the host”^[119]^. One step further, the term “psychobiotics” has recently emerged to describe probiotics that confer improvements on mental health, enabling more acute dissection of microbial impacts on cognition, learning, memory, and behavior from those on general health^[120,121]^.

**Table 1:** Efficacies of probiotics and their neurological impacts during clinical and animal studies

**Probiotics used**	**Study model**	**Disorder**	**Effect**	**References**
Strains belonging to the species: *L. acidophilus*, *L. casei*, *B. bifidum*, and* L. fermentum*	Clinical	AD	Improvement in cognition, shifts in biochemical measurements in the serum	Akbari *et al.*^[129]^
Strains belonging to the species: *L. acidophilus*, *B. bifidum*, and *B. longum* with selenium supplementation	Clinical	AD	Probiotic co-supplementation improves cognitive and serum biochemical measures compared to placebo or only selenium groups	Tamtaji *et al.*^[131]^
ProBiotic-4 (strains belonging to the species: *B. animalis* subsp.* lactis*, *L. casei*, *B. bifidum*, and *L. acidophilus*)	Mouse	AD	Improves memory deficits, neuronal and synaptic injuries, glial activation, protects from leaky gastrointestinal and BBB, and lowers proinflammatory cytokine levels	Yang *et al.*^[87]^
*L. helveticus* R0052 and *B. longum *R0175	Clinical and rat	Anxiety	Probioitic formulation of *L. helveticus* and *B. longum* reduced anxiety in rats and psychological distress in humans	Messaoudi *et al.*^[123]^
Visbiome® (*L. paracasei *DSM 24733, *L. plantarum *DSM 24730, *L. acidophilus *DSM 24735, *L. delbruckei *subsp. *bulgaricus *DSM 24734, *B. longum *DSM 24736, *B. longum *subsp.* infantis* DSM 24737, *B. breve *DSM 24732, and *S. thermophilus *DSM 24731)	Clinical	ASD	In non-gastrointestinal ASD patients: improvements in Total Autism Diagnostic Observation Schedule (ADOS) Calibrated Severity Score and social affect; In ASD patients with gastrointestinal symptoms, improvements in gastrointestinal symptoms, adaptive functioning, and multisensory processing	Santocchi *et al.*^[125]^
Strains belonging to the species: *L. acidophilus*, *B. bifidum*, *L. reuteri*, and* L. fermentum*	Clinical	PD	Randomized, double-blind, placebo-controlled study with 60 PD patients. Probiotics decreased Movement Disorders Society-Unified Parkinson’s Disease Rating Scale (MDS-UPDRS). Probiotics also lowered high-sensitivity C-reactive protein and malondialdehyde levels, with elevations in glutathione levels	Tamtaji *et al.*^[135]^
Strains belonging to the species: *L. acidophilus*, *B. bifidum*, *L. reuteri*, and* L. fermentum*	Clinical	PD	Randomized, double-blind, placebo-controlled study with 50 PD patients. 12-week probiotic treatment lowered IL1, IL8, and TNFα levels in peripheral blood mononuclear cells (PBMC). TGFβ and PPARγ levels were upregulated in PBMC	Tamtaji *et al.*^[136]^
Visbiome® (*L. paracasei *DSM 24733, *L. plantarum *DSM 24730, *L. acidophilus *DSM 24735, *L. delbruckei *subsp. *bulgaricus *DSM 24734, *B. longum *DSM 24736, *B. longum *subsp.* infantis* DSM 24737, *B. breve *DSM 24732, and *S. thermophilus *DSM 24731)	Clinical	MS	Nine MS patients were treated with probiotics for two months. Certain taxa known to be depleted in MS, such as *Lactobacillus*, were restored by probiotic treatment. MS-associated dysbiosis was attenuated by probiotics. Probiotics caused an antiinflammatory immune reaction particular to antigen-presenting cells	Tankou *et al.*^[146]^
*L. reuteri *ATCC PTA 6475	Mouse	ASD	*L. reuteri *or tetrahydrobiopterin (BH4) can improve sociability in the *Cntnap2^-/-^* mice through enhanced social-reward circuitry, but not hyperactivity	Buffington *et al.*^[72]^
*L. reuteri *ATCC PTA 6475	Mouse	ASD	*L. reuteri *protects from abnormal social behavior in genetic, environment, and idiopathic mouse models for ASD through a vagus nerve and oxytocin-dependent manner	Sgritta *et al.*^[114]^
*L. reuteri *ATCC-PTA-6475	Mouse	ASD	*L. reuteri *improves social behavior and social memory through correcting oxytocin levels in maternal high-fat diet-induced ASD offspring	Buffington *et al.*^[9]^
*L. reuteri *RC-14	Mouse	ASD	In the BTBR genetic mouse model for ASD, *L. reuteri* treatment improves sociability and repetitive behavior through reduced intestinal permeability	Nettleton *et al.*^[122]^
*Clostridia*-dominant spore-forming bacteria	Mouse	Abnormal neurodevelopment	Colonization of ABX mice with *Clostridia-*dominant spore-forming bacteria improves axonogenesis and sensorimotor behavior	Vuong *et al.*^[74]^
*Bacteroides fragilis *NCTC 9343	Mouse	ASD	*B. fragilis *treatment in MIA offspring improves communication, repetitive, anxiety, and sensorimotor behaviors through modulation of serum metabolites but not social behaviors	Hsiao *et al.*^[106]^
*E. faecalis *ATCC 19433	Mouse	ASD	*E. faecalis* treatment in ABX mice modulates the HPA axis and reduces social stress-induced corticosterone levels to promote sociability	Wu *et al.*^[4]^
*L. rhamnosus *JB-1	Mouse	Anxiety	*L. rhamnosus *JB-1 treatment alters GABA_B1b_ expression and corticosterone levels to reduce anxiety and depression-associated behaviors	Bravo *et al.*^[81]^
*B. dentium *ATCC 27678	Mouse	ASD	*B. dentium* treatment in GF mice protects from repetitive behavior and high anxiety through increased serotonin signaling	Engevik *et al.*^[76]^
*B. bifidum*, *B. longum*, *L. rhamnosus*, *L rhamnosus GG*, *L. plantarum LP28*, and* L. lactis *subsp*. Lactis*	Mouse	PD	In the transgenic MitoPark PD mouse model, probiotic treatment improves motor functions in gait pattern, balance, and coordination. Tyrosine hydroxylase expressing neurons in the substantia nigra are protected following probiotics treatment	Hsieh *et al.*^[132]^
*L. rhamnosus GG*, *B. animalis *subsp.* lactis *BB-12, and *L. acidophilus *LA-5	Mouse	PD	Probiotics protected from neurotoxicity in 1-methyl-4-phenyl-1,2,3,6-tetrahydropyridine PD model. In the rotenone-induced PD model, probiotics increased neurotrophic factors and butyrate	Srivastav *et al.*^[133]^
Slab51® (*S. thermophilus* DSM 32245, *B. lactis* DSM 32246, *B. lactis* DSM 32247, *L. acidophilus* DSM 32241, *L. helveticus* DSM 32242, *L. paracasei* DSM 32243, *L. plantarum* DSM 32244, and *L. brevis* DSM 27961	Mouse	PD	Slab51® improves asymmetrical motor performance. Slab51® maintains tyrosine hydroxylase levels in dopaminergic neurons during PD induction. Probiotics further protect from microglia and astrocyte reactivity. BDNF and PPARγ levels are also protected by Slab51®	Castelli *et al.*^[134]^
*L. paracasei *DSM 13434, *L. plantarum *DSM 15312, and DSM 15313	Mouse	MS	In EAE mice, probiotics reduced CNS inflammation through Treg induction within the mesenteric lymph nodes. TGFβ1, IL27, and IL10 were also increased	Lavasani *et al.*^[145]^

Clinical studies utilizing probiotics have been addressed, with details on strain information, neurological conditions improvements, and biomarkers correlations. Animal studies featuring probiotic intervention have also been included and present potential mechanisms of action to improve behavior. AD: Alzheimer’s disease; ASD: autism spectrum disorder; PD: Parkinson’s disease; MS: multiple sclerosis; ABX: antibiotic-treated; MIA: maternal immune activation; GF: germ-free.

Probiotics including *L. reuteri *ATCC PTA 6475, *L. rhamnosus *JB-1, and *Bifidobacterium longum *R0175 are well-studied psychobiotic due to their ability to reverse social deficits and high anxiety levels in mice^[9,81,114,122,123]^. Such evidence has been translated into clinical studies of microbes as therapeutics for ASD patients. A study in Egypt tested a combination of *L. acidophilus*, *L. rhamnosus*, and *B. longum* in 30 children with ASD and found improvements in both autism severity as well as gastrointestinal symptoms (GS)^[124]^. Interestingly, psychobiotic efficacy may depend on the severity of GS among ASD patients. A recent study in Italy utilized a patented mixture (Visbiome®) of* L. paracasei *DSM 24733, *L. plantarum *DSM 24730, *L. acidophilus *DSM 24735, *Lactobacillus delbruckei *subsp. *bulgaricus *DSM 24734, *B. longum *subsp. *infantis *DSM 24736, *Bifidobacterium longum *subsp.* infantis *DSM 24737, *Bifidobacterium breve *DSM 24732, and *Streptococcus thermophilus *DSM 24731 found more significant improvements in adaptive functioning, developmental pathways, sensory processing, and gastrointestinal function among ASD patients with GS compared to those without^[125]^. This harkens back to the theory that ASD, as a complex spectrum of behavioral phenotypes, may have many pathological mechanisms, among which microbiota intervention can effectively target^[72]^.

Besides probiotics, clinical studies utilizing FMT further championed the use of gut microbes as therapeutics for ASD. A four-week FMT treatment utilizing the Standard Human Gut Microbiota (SHGM) in 40 children with ASD demonstrated improvements to GS, behavioral ASD symptoms, and normalization of microbiome signatures towards those of control children^[126]^. A separate clinical trial with 18 ASD patients and an eight-week FMT treatment period using SHGM also corroborate these therapeutic efficacies^[127]^. Surprisingly, these improvements were observed two years following the initial study, indicating long-term benefits to microbiota-mediated therapeutics^[128]^.

### Probiotic therapeutics for neurodegenerative disorders

Clinical studies have also identified microbes capable of therapeutic efficacy in neurodegenerative disorders. One study with 60 AD patients revealed that a mixture of strains from *L. acidophilus*, *L. casei*,* B. bifidum*, and *Lactobacillus fermentum *could improve cognitive function and metabolic levels^[129]^. However, in a follow-up study, the group later tested another combination of probiotics composed of *L. fermentum*,* L. plantarum*,* B. animalis *subsp.* lactis*,* L. acidophilus*,* B. bifidum*, and *B. longum*, which was incapable of improving memory scores, suggesting that the specific probiotic composition and severity of AD must be taken into account^[130]^. Probiotics are also effective in co-supplementation regimen, as a consortium of *L. acidophilus*,* B. bifidum*, and *B. longum* with selenium supplementation could enhance cognitive and metabolic profiles compared to selenium only or placebo control groups^[131]^.

Parkinson’s disease (PD) is another neurodegenerative disorder that affects neuromotor functions. Evidence on the efficacy of probiotic treatment on PD is limited. Still, one recent preclinical mouse study suggests that mixtures containing *B. bifidum*, *B. longum*,* L. rhamnosus*,* L. rhamnosus GG*, *L. plantarum *LP28, and *Lactococcus lactis *subsp. *lactis *may improve motor coordination and performance^[132]^. Similar improvements in motor behavior protection from neurotoxicity were observed with probiotic cocktails containing *L. rhamnosus GG*,* B. animalis *subsp.* lactis* BB-12, and *L. acidophilus *LA-5, as well as the commercial formulation Slab51®, which includes *S. thermophilus *DSM 32245,* B. animalis *subsp. *lactis *DSM 32246,* B. animalis *subsp.* lactis *DSM 32247, *L. acidophilus *DSM 32241, *Lactobacillus helveticus *DSM 32242, *L. paracasei *DSM 32243, *L. plantarum *DSM 32244*, *and *Lactobacillus brevis *DSM 27961^[133,134]^. Clinical studies are also beginning to reveal the potential benefits of probiotic treatment on PD. A study featuring 60 PD patients who underwent 12 weeks of probiotic treatment containing a mixture of *L. acidophilus*,* B. bifidum*,* L. reuteri*, and *L. fermentum* reported improvements in motor function^[135]^. The same group identified this probiotic treatment to lower gene expression in inflammatory cytokines, including IL1, IL8, and TNFα, indicating an immune-mediated effect of probiotics during PD^[136]^.

### Immune-mediated probiotic efficacy

Interestingly, many of these probiotics utilized in neurological disorders also have immunological benefits, leading to the hypothesis that probiotic intervention may alleviate neurological symptoms through the host immune pathway. For example, strains of *B. longum *subsp. *infantis*, including* B. longum *subsp. *infantis *35624 may affect both immunological and neurological pathways, as it is demonstrated to attenuate serum and brain IFNγ, TNFα, and IL6 levels, as well as to increase the serotonin precursor tryptophan in the plasma and PSD-95 and BDNF levels in the brain^[137,138]^. This is highlighted in GF mice, where mono-colonization with *B. longum *subsp. *infantis* can protect them from exaggerated hypothalamic-pituitary-adrenal stress responses^[1]^. Furthermore, combination therapy of *L. acidophilus *ATCC 53544 and *B. longum *subsp. *infantis* ATCC 15697 during pregnancy protects offspring mice from systemic and neuroinflammation, leaky BBB, and astrocyte and microglia activation^[139]^. Analyses of the gastrointestinal tract in these offspring reveal a more robust intestinal integrity that translated to reduced serum IL1β, TNFα, and IL6 circulation, elucidating the multifaceted beneficial capacity of probiotics^[140]^.

Moreover, certain probiotics such as *L. acidophilus *LA257,* L. reuteri *ATCC 23272, and *B. bifidum *PRI1 are Treg inducers capable of suppressing inflammation in numerous disease models^[14,141,142]^. This is demonstrated in neuroinflammatory and neurological disorders. SCFA from microbes, such as the Treg-inducing butyrate, can alleviate EAE by attenuating IL17 levels^[143,144]^. Likewise, a mixture of *L. paracasei *DSM 13434, *L. plantarum *DSM 15312, and DSM 15313 also induce Treg cells and IL10, leading to improvements in EAE^[145]^. Clinically, the Visbiome® mixture has been shown to protect from MS-associated inflammation^[146]^. While it is yet to be displayed, it is also possible that microbiome-enhanced serotonin levels may activate brain-resident Treg cells expressing 5-HT7. In such a manner, probiotics targeting immune regulation may be an effective strategy for alleviating neurological disorders, especially those driven by neuroinflammation. With advances in the field, we anticipate future publications to better reveal the centrality of brain-resident immune cells in mediating the gut-brain axis.

## CONCLUSION

The gut-brain axis is an emerging interdisciplinary field that harbors a prominent capacity to change how modern biomedicine approach neurodevelopment and neurological disorders. Identifying unique immune populations within the brain, such as T cells, demonstrates that the brain is not an isolated environment as it once was believed to be but is dynamically shaped by external factors. These CNS-associated T cells can shift the brain towards a proinflammatory or antiinflammatory state through close interactions with neurons that directly express cytokine receptors, microglia, and the BBB^[19,55,147]^. Brain-resident Tregs are a newly discovered subset of T cells that regulate neuroinflammation and neurological recovery following stroke^[20]^. Expressing distinct nervous system-related genes such as serotonin receptors and brain Tregs may have undiscovered tissue-specific roles within the CNS^[20]^.

First alluded to by comorbidities between gastrointestinal and neurological disorders, evidence from the recent decade indicates a close link between the gut microbiota, host immunity, and the CNS. Indeed, GF and ABX mouse studies have demonstrated that the absence of microbiome results in abnormal neurodevelopment and behaviors often associated with ASD^[73]^. Furthermore, from ASD to AD, numerous animal and clinical studies in recent years have unveiled the ability of microbiota interventions to modulate neuroinflammation and neurotransmitter levels. Probiotics have shown efficacy in improving cognition and behaviors among ASD and AD patients, and further studies into their mechanisms may lead to more comprehensive strategies for targeted therapeutics^[124,129]^. It is necessary to note that probiotic strains differ in their ability to impact the immune system and neurological outcomes^[148]^. Thus, further strain-specific studies of probiotics are necessary to identify their psychobiotic efficacies and mechanisms of action. 

Gut immune systems play a crucial role in maintaining the homeostasis of the gut microenvironment and act as the intermediaries to the systemic impacts of gut microbes on the host. *In vivo *experiments in mice hint that gut microbes may even regulate immune populations within the CNS. The absence of gut microbes in GF and ABX mice results in a leaky BBB, possibly allowing the enhanced infiltration of peripheral immune populations into the CNS^[85]^. On the other hand, probiotics can strengthen the BBB integrity and protect from neuroinflammation^[88]^. It will be interesting to see whether barrier integrity between the intestinal tract and the BBB is correlated. Microbes and their SCFAs can also influence the functional characteristics of microglia and lymphocytes within the CNS, determining the fate of neurological disorders driven by neuroinflammation.

We thus hypothesize that the host immunity is a critical mediator within the gut-brain axis and propose the expansion of this field into the gut-immune-brain axis. With further research, a broader understanding of the capacity in which immune populations of the CNS reconcile gut microbes with neurodevelopmental and neuroinflammatory pathways may provide new avenues for effective therapeutic interventions utilizing probiotics.

## DECLARATIONS

### Copyright

© The Author(s) 2022.
